# Hamiltonian Monte Carlo sampling to estimate past population dynamics using the skygrid coalescent model in a Bayesian phylogenetics framework

**DOI:** 10.12688/wellcomeopenres.15770.1

**Published:** 2020-03-30

**Authors:** Guy Baele, Mandev S. Gill, Philippe Lemey, Marc A. Suchard

**Affiliations:** 1 Department of Microbiology, Immunology and Transplantation, Rega Institute, KU Leuven, Herestraat 49, 3000, Leuven, Belgium; 2 Departments of Biostatistics, Biomathematics and Human Genetics, University of California, Los Angeles, 695 Charles E. Young Drive, Los Angeles, California, 90095-1766, USA

**Keywords:** Hamiltonian Monte Carlo, Markov chain Monte Carlo, Bayesian skygrid, phylogenetics, pathogen phylodynamics, BEAST, BEAGLE

## Abstract

Nonparametric coalescent-based models are often employed to infer past population dynamics over time. Several of these models, such as the skyride and skygrid models, are equipped with a block-updating Markov chain Monte Carlo sampling scheme to efficiently estimate model parameters. The advent of powerful computational hardware along with the use of high-performance libraries for statistical phylogenetics has, however, made the development of alternative estimation methods feasible. We here present the implementation and performance assessment of a Hamiltonian Monte Carlo gradient-based sampler to infer the parameters of the skygrid model. The skygrid is a popular and flexible coalescent-based model for estimating population dynamics over time and is available in BEAST 1.10.5, a widely-used software package for Bayesian pylogenetic and phylodynamic analysis. Taking into account the increased computational cost of gradient evaluation, we report substantial increases in effective sample size per time unit compared to the established block-updating sampler. We expect gradient-based samplers to assume an increasingly important role for different classes of parameters typically estimated in Bayesian phylogenetic and phylodynamic analyses.

## Introduction

Inference of effective population size over time from a sample of molecular sequences is a key aspect of many phylodynamics studies. Inference methods typically employ coalescent models that connect population dynamics to the shape of a genealogy relating such a sample. A wide range of coalescent models has been developed over the past decades, gradually extending the original coalescent theory of Kingman ^1^. In particular, flexible nonparametric coalescent models have become widely used ^2– 8^. These models typically posit that the effective population size as a function of time (also referred to as the “demographic function”) assumes a piecewise constant form, thereby avoiding restrictive *a priori* assumptions about the specific parametric form of the demographic function. In a Bayesian framework, coalescent models function as prior distributions for phylogenetic trees and, in conjunction with observed sequence data likelihoods based on continuous-time Markov models for molecular character evolution on trees ^9^, they enable the estimation of effective population size directly from molecular sequence data.

Among such nonparametric models, the Bayesian skygrid ^7^ has emerged as a popular choice for a number of reasons. Unlike most competing models, the skygrid can incorporate data from multiple unlinked genetic loci, which has proven to be invaluable for accurate reconstruction of past population dynamics ^10^. Further, the skygrid has been extended to integrate external time-varying covariates ^11^, enabling the assessment of the relationship between effective population size and ecological and epidemiological indices, and also potentially yielding improved estimates of effective population size trajectories and genealogies ^12^. Like the skyride model ^6^, the skygrid aims to construct a smooth population size trajectory over time through a Gaussian Markov random field (GMRF) smoothing prior. Finally, the skygrid is implemented in the widely used BEAST 1.10 software package ^13, 14^, where it can be combined with a wide range of models for evolutionary heterogeneity, phylogeography, and phenotypic trait evolution to build sophisticated phylodynamic models. This enables the efficient analysis of large data sets using a combination of complex models, in large part owing to BEAST’s integration with BEAGLE, a high-performance library for efficient phylogenetic likelihood calculation ^15^.

Inference in Bayesian phylogenetics relies on Markov chain Monte Carlo (MCMC) methods to sample from the posterior distribution ^16, 17^. MCMC methods simulate a correlated sample that converges in distribution to the posterior. The efficiency of a given MCMC algorithm depends on the transition kernel, which proposes a new simulated value based upon the current simulated value. Standard random walk transition kernels propose new values in a relatively blind fashion and generally update only one component of the multi-dimensional parameter space at a time. This can lead to a very slow, inefficient exploration of the posterior distribution where the MCMC algorithm would have to run for a relatively large number of iterations in order to simulate a suitable sample.

Fortunately, sophisticated, tailor-made transition kernels can often sample from the posterior much more efficiently. Made possible by the specific structure of the model, the skygrid adapts a highly efficient block-updating MCMC (BUMCMC) sampling scheme ^18^ that simultaneously proposes new values for the GMRF precision parameter and the effective population size values that correspond to the different levels of the piecewise constant demographic function. The GMRF smoothing prior along with the nonparametric coalescent likelihood gives the full conditional density of the effective population size in the form of a hidden Markov random field, allowing us to efficiently sample from its Gaussian approximation ^19^.

Hamiltonian Monte Carlo (HMC) ^20, 21^ is an MCMC sampling scheme that bears some similarities to the BUMCMC sampler in that it aims to efficiently explore high probability regions of the posterior distribution and update all dimensions of the model parameter space simultaneously. HMC proceeds by introducing fictitious auxiliary “momentum” variables and reduces simulating from the posterior distribution to a matter of tracing Hamiltonian dynamics. While HMC’s theoretical basis in differential geometry initially hindered its adoption, it has emerged in recent years as a widely-used method in statistical computing ^22^. While adapting HMC to optimize the search through tree space is currently not possible, Dinh *et al.* ^23^ have developed an approach to sample from distributions on spaces with intricate combinatorial structure (such as for phylogenetic tree space). Applications of HMC in the field of Bayesian phylogenetics have started to emerge that focus on efficiently estimating classes of model parameters. Recently, Ji *et al.* ^24^ developed a linear-time algorithm for *O*( *N*)-dimensional gradient evaluation – where *N* is the number of sampled molecular sequences – and showed HMC to greatly improve inference efficiency of branch-specific evolutionary rates.

Here, we present the implementation of an HMC transition kernel for the nonparametric skygrid coalescent model and compare its performance to the BUMCMC sampler. We compare the performance on three real viral data sets and find that in all cases HMC more efficiently explores the posterior distribution of skygrid model parameters. In some instances, the improvement afforded by HMC is especially striking, generating effectively independent posterior samples over five times as fast as BUMCMC.

## Methods

### The skygrid nonparametric coalescent model

The skygrid posits that demographic function *N _e_*( *t*) is a piecewise constant function that can change values only at pre-specified points in time known as “grid points.” As in Gill *et al.* ^7^, let *x*_1_,…, *x _M_* denote the temporal grid points, where *x*_1_ *≤ x*_2_ *≤ · · · ≤ x _M −_*_1_ *≤ x _M_* . The *M* grid points divide the demographic history timeline into *M* + 1 intervals so that the demographic function is fully specified by a vector ****θ**** = ( *θ*_1_, …, *θ _M_*_+1_) of values that it assumes on those intervals. Here, *N _e_*( *t*) = *θ _k_* for *x* *_k−_*_1_ *≤ t < x _k_*, *k* = 1, . . ., *M*, where it is understood that *x*_0_ = 0. Also, *N _e_*( *t*) = *θ* *_M_*_ +1_ for *t ≥ x _M_* . Note that *x _M_* is the time furthest back into the past at which the effective population size can change. The values of the grid points as well as the number *M* of total grid points are specified beforehand by the user. A typical way to select the grid points is to decide on a resolution *M*, let *x _M_* assume the value furthest back in time for which the data are expected to be informative, and space the remaining grid points evenly between *x*_0_ = 0 and *x _M_*. Alternatively, as discussed in the next section, grid points can be selected to align with covariate sampling times in order to facilitate the modeling of associations between the log effective population size and external covariates.

Suppose we have *m* known genealogies *g*_1_, …, *g _m_* representing the ancestries of samples from *m* separate genetic loci with the same effective population size *N _e_*( *t*). We assume *a priori* that the genealogies are independent given *N _e_*( *t*). This assumption implies that the genealogies are unlinked which commonly occurs when researchers select loci from whole genome sequences or when recombination is very likely, such as between genes in retroviruses. The likelihood of the vector **g** = ( *g*_1_,…, *g _m_*) of genealogies can then be expressed as the product of likelihoods of individual genealogies:

$$P(\text{g}|\theta )\;=\;\prod_{i=1}^{m}P(g_{i}\text{|}\;\theta ).\;(1)$$

To construct the likelihood of genealogy *g _i_*, let *t*_0 _*i*__ be the most recent sampling time of sequences contributing to genealogy *i* and *t*_MRCA _*i*__ be the time of the MRCA for locus *i*. Let *x _α _i__* denote the minimal grid point greater than at least one sampling time in the genealogy, and *x _β _i__* the greatest grid point less than at least one coalescent time. Let *u _ik_* = [ *x _k–1_*, *x _k_*], *k* = *α _i_* + 1, …, *β _i_*, *u _iα _i__* = [ *t _0 _i__*, *x _α _i__*], and *u*_*i*( *β _i_* + 1)_ = [ *x _β _i__*, *t*_MRCA _*i*__]. For each *u _ik_* we let *t _kj_*, *j* = 1,…, *r _k_*, denote the ordered times of the grid points and sampling and coalescent events in the interval. With each *t _kj_* we associate an indicator *Φ _kj_* which takes a value of 1 in the case of a coalescent event and 0 otherwise. Finally, let *v _kj_* denote the number of lineages present in the genealogy in the interval [ *t _kj_*, *t _k( j+1)_*]. Following Griffiths and Tavaré ^25^, the likelihood of observing an interval is

$$\begin{array}{l} P(u_{ik}|\theta_{k})=\;\prod_{1\leq j<r_{k}:\Phi_{kj}=1}\frac{v_{kj}(v_{kj}–1)}{{2\theta }_{k}}\\ \;\times \;\prod_{j=1}^{r_{k}–1}\exp\left[–\frac{v_{kj}(v_{kj}–1)(t_{k(j+1)}–t_{kj})}{{2\theta }_{k}}\right] \end{array}\;(2)$$

for *k* = *α*_*i*_ *β*_*i*_ + 1.

The product of interval likelihoods ( 2) yields the likelihood of coalescent times given the sampling times with genealogy *g _i_*. To obtain the likelihood of the genealogy, however, we must account for the specific lineages that merge and result in coalescent events. Let P _*_( *u _ik_*| *θ _k_*) denote *P*( *u _ik_*| *θ _k_*) except with factors of the form $\frac{v_{kj}(v_{kj}–1)}{{2\theta }_{k}}$ replaced by $\frac{2(2–1)}{{2\theta }_{k}}\;=\;\frac{1}{\theta_{k}}.$ Then

$$P(g_{i}\text{|}\theta )=\prod_{k=\alpha_{i}}^{\beta_{i}+1}P_{*}(u_{ik}|\theta_{k}).\;(3)$$

We introduce some notation that will facilitate the derivation of a Gaussian approximation used to construct an MCMC transition kernel. If *c _ik_* denotes the number of coalescent events which occur during interval *u _ik_*, we can write

$$P(g_{i}\text{|}\theta )=\prod_{k=\alpha_{i}}^{\beta_{i}+1}{\left(\frac{1}{\theta_{k}}\right)}^{c_{ik}}\exp\left[–\frac{S_{ik}}{\theta_{k}}\right],\;(4)$$

where the *S _ik_* are sufficient statistics from the genealogy. Rewriting this expression in terms of *γ _k_* = log( *θ _k_*), we arrive at

$$P(g_{i}\text{|}\gamma )=\prod_{k=\alpha_{i}}^{\beta_{i}+1}e^{–\gamma_{k}c_{ik}}\exp\left[–S_{ik}e^{–\gamma_{k}}\right]\;(5)$$

$$\;=\prod_{k=\alpha_{i}}^{\beta_{i}+1}\exp\left[–\gamma_{k}c_{ik}–S_{ik}e^{–\gamma_{k}}\right].\;(6)$$

Invoking conditional independence of genealogies, the likelihood of the vector **g** of genealogies is

$$P(\text{g}|\gamma )=\prod_{i=1}^{m}P(g_{i}\text{|}\gamma )\;(7)$$

$$=\prod_{i=1}^{m}\prod_{k=\alpha_{i}}^{\beta_{i}+1}\exp\left[–\gamma_{k}c_{ik}–S_{ik}e^{–\gamma_{k}}\right]\;(8)$$

$$=\exp\left[\sum_{k=1}^{M+1}\left[–\gamma_{k}c_{k}–S_{k}e^{–\gamma_{k}}\right]\right]\;(9)$$

where $c_{k}\;=\;\sum_{i=1}^{m}c_{ik}$ and $S_{k}\;=\;\sum_{i=1}^{m}S_{ik}$; here, *c _ik_* = *S _ik_* = 0 if *k* ∉ [α _i_, β _i_ + 1].

The skygrid incorporates the prior assumption that effective population size changes continuously over time by placing a GMRF prior on ***γ***:

$$P\left(\gamma |\tau \right)\propto \tau^{M/2}\exp\left[–\frac{\tau }{2}\sum_{i=1}^{M}{\left(\gamma_{i+1}–\gamma_{i}\right)}^{2}\right].\;(10)$$

This prior does not inform the overall level of the effective population size, just the smoothness of the trajectory. One can think of the prior as a first-order unbiased random walk with normal increments. The precision parameter *τ* determines how much differences between adjacent log effective population size values are penalized. We assign *τ* a gamma prior:

$$P(\tau )\propto \tau^{a–1}e^{–b\tau }.\;(11)$$

In absence of prior knowledge about the smoothness of the effective population size trajectory, we choose *a* = *b* = 0.001 so that it is relatively uninformative. Conditioning on the vector of genealogies, we obtain the posterior distribution

P(γ,τ|g)∝P(g|γ)P(γ|τ)P(τ).(12)

### Block-updating Markov chain Monte Carlo sampling

The original implementation of the skygrid adapts a BUMCMC sampling scheme for GMRFs ^18^ to sample ***γ*** and *τ* when approximating the posterior ( 12). First, consider the full conditional density

$$\begin{array}{l} P\left(\gamma |\text{g},\tau \right)\propto P\left(\text{g}|\gamma \right)P\left(\gamma |\text{τ}\right)\\ \;\propto \tau^{M/2}\exp\left[-\frac{\tau }{2}\gamma^{\prime }\text{Q}\gamma -\sum_{k=1}^{M+1}\left(\gamma_{k}c_{k}+S_{k}e^{-\gamma_{k}}\right)\right]. \end{array}\;(13)$$

Let *h _k_* ( *γ _k_*) = ( *γ _k_c _k_* + *S _k_* *e ^−γ _k_^*). We can approximate each term *h _k_* ( *γ _k_*) by a second-order Taylor expansion about, say, ${\hat{\gamma }}_{k}$:

$$\begin{array}{l} h_{k}(\gamma_{k})\approx \;\;h_{k}({\hat{\gamma }}_{k})+{h^{\prime }}_{k}({\hat{\gamma }}_{k})(\gamma_{k}–{\hat{\gamma }}_{k})+\;\frac{1}{2}{h^{\prime \prime }}_{k}({\hat{\gamma }}_{k})(\gamma_{k}–{\hat{\gamma }}_{k}{)}^{2}\\ \;=\;S_{k}e^{–{\hat{\gamma }}_{k}}\left(\frac{1}{2}{{\hat{\gamma }}_{k}}^{2}\;+\;{\hat{\gamma }}_{k}\;+\;1\right)\\ \;+\left[c_{k}–\;S_{k}e^{–{\hat{\gamma }}_{k}}–\;S_{k}e^{–{\hat{\gamma }}_{k}}\;{\hat{\gamma }}_{k}\right]\gamma_{k}\\ \;+\left[\frac{1}{2}S_{k}e^{–{\hat{\gamma }}_{k}}\right]\gamma_{k}^{2}. \end{array}\;(14)$$

We center the Taylor expansion about a point $\hat{\gamma }=({\hat{\text{γ}}}_{1},...,{\hat{\text{γ}}}_{M+1})$ obtained iteratively by the Newton-Raphson method:

$$\gamma_{\left(n+1\right)}=\;\gamma_{\left(n\right)}\;–\;{\left[d^{2}f(\gamma_{\left(n\right)})\right]}^{-1}(df(\gamma_{\left(n\right)}){)}^{\prime }\;(15)$$

with ***γ***_(0)_ = ***γ*** ^(n)^ the current value of ***γ***. Here,

$$f\left(\gamma \right)=-\frac{1}{2}\gamma^{\prime }\tau \text{Q}\gamma -\sum_{k=1}^{M+1}\left(\gamma_{k}c_{k}+S_{k}e^{-\gamma k}\right).\;(16)$$

Replacing the terms *h _k_* ( *γ _k_*) with their Taylor expansions yields the following second-order Gaussian approximation to the full conditional density *P*( ***γ|*** **g**, *τ*) :

$$\begin{array}{l} P\left(\gamma |\text{g},\tau \right)\approx \tau^{M/2}\text{exp}\left[-\frac{1}{2}\gamma^{\prime }\left[\tau \text{Q}+\text{Diag}\left(S_{k}e^{-{\hat{\gamma }}_{k}}\right)\right]\right]\\ \;-\text{exp}\left[\sum_{k=1}^{M+1}\left(c_{k}-S_{k}e^{-{\hat{\gamma }}_{k}}-S_{k}e^{-{\hat{\gamma }}_{k}}{\hat{\gamma }}_{k}\right)\gamma_{k}\right], \end{array}\;(17)$$

where Diag( *·*) is a diagonal matrix.

Starting from current parameter values ( ***γ*** ^( *n*)^, *τ* ^( *n*)^), we first generate a candidate value for the precision, τ ^*^ = *τ* ^( *n*)^ *f*, where *f* is drawn from a symmetric proposal distribution with density $P(f)\;\propto \;f+\frac{1}{f}$ defined on [1 */F*, *F*]. The tuning constant *F* controls the distance between the proposed and current values of the precision. Next, conditional on τ ^*^, we propose a new state γ ^*^ using the Gaussian approximation ( 17) to the full conditional density *P*( *γ|* **g**, τ ^*^). In the final step, the candidate state ( τ ^*^, γ ^*^) is accepted or rejected according to the Metropolis-Hastings ratio ^16, 17^.

### Hamiltonian Monte Carlo sampling

HMC can be applied to most problems with continuous parameter spaces and produces distant proposals for the Metropolis algorithm ^16^ that nevertheless enjoy a high probability of acceptance. This enables efficient MCMC sampling by avoiding the slow exploration of the state space that accompanies simple random-walk proposals. Consider a *d*-dimensional *position* vector ****Φ****. This is the parameter whose posterior distribution we wish to sample from, and in the case of the skygrid, we have ****Φ**** = ( ****γ****, *τ*, ***β***). HMC proceeds by introducing a *d*-dimensional vector of auxiliary *momentum* variables **p** and sampling from the product distribution *π*( ****Φ****, **p**) = *π*( ***Φ***) *π*( **p**) by simulating Hamiltonian dynamics. The Hamiltonian function is defined as

H(Φ,p)=U(Φ)+K(p),(18)

where *U*( ****Φ****), the *potential energy*, is defined as the negative log density of the position vector ****Φ**** and *K*( **p**), the *kinetic energy* of the momentum variable **p** is defined as *K*( **p**) = **p** *^T^* **M** ^−1^ **p** */*2, where **M** is a symmetric, positive definite (usually diagonal) matrix known as the “mass matrix.” For **p**, we make the common assumption that it follows a multivariate normal distribution **p** *~ N*( **0**, **M**). It has become standard in basic HMC implementations to set **M** = **I**, but we will discuss a more informed choice later.

HMC generates a Metropolis proposal from the current state ( ****Φ****_0_, **p**_0_) in the space ( ****Φ****, **p**) that evolves according to Hamilton’s equations:

$$\begin{array}{l} \frac{\text{d}\text{p}}{\text{d}t}=-\nabla U\left(\Phi \right)=\nabla \log\pi \left(\Phi \right)\\ \frac{\text{d}\Phi }{\text{d}t}=\nabla K\left(\text{p}\right)={\text{M}}^{-1}\text{p}. \end{array}\;(19)$$

The *leapfrog* method to approximate a solution to Equation 19 performs the following updates for each of *n* leapfrog steps of size :

$$\begin{array}{l} {\text{p}}_{t+/2}={\text{p}}_{t}+\frac{}{2}\nabla \log\pi \left(\Phi_{t}\right)\\ \Phi_{t+}=\Phi_{t}+M^{-1}{\text{p}}_{t+/2}\\ {\text{p}}_{t+}={\text{p}}_{t+/2}+\frac{}{2}\nabla \log\pi \left(\Phi_{t+}\right). \end{array}(20)$$

The use of HMC therefore requires the user to specify these two parameters, i.e. the step size ** and the number of steps *n*. In addition, we assume a standard HMC transition kernel by employing an identity matrix **I** for the mass matrix **M**. Through its internal auto-tuning capabilities, BEAST 1.10 enables tuning ** during an ongoing analysis, but *n* still needs to be provided by the user.

### Data

We compare the performance of the BUMCMC and HMC transition kernels for the skygrid on three real data sets. First, we analyse the population dynamics of the rabies epizootic among raccoons in the northeastern United States starting in the late 1970s ^26^. The sequence data consist of 47 sequences sampled from rabid raccoons between 1982 and 2004 and encompass the complete rabies nucleoprotein (N) genes as well as large portions of the glycoprotein (G) genes. Based on a previous analysis ^11^, we set a cutoff for the skygrid of 40 years during which we estimate the log population size for 50 time intervals. We assume a single HKY nucleotide substitution model ^27^ while accounting for among-site rate variation ^28^, and assume an uncorrelated relaxed molecular clock with an underlying lognormal distribution ^29^.

The second data set consists of 196 Ebola virus (EBOV) sequences originating from Sierra Leone, previously analysed in Hill and Baele ^14^. Based on information obtained from a large-scale study of the West African Ebola virus outbreak in 2013–2016 ^30^, we set the cutoff for the skygrid to one year, and we estimate the log population size for each week for a total of 52 log population size estimates. We partition the coding part of the data set according to codon position, and create a fourth data partition containing the aggregated intergenic region data ^30^. For each of the four resulting partitions, we assume an HKY nucleotide substitution model ^27^ while accounting for among-site rate variation ^28^. We assume an uncorrelated relaxed molecular clock with an underlying lognormal distribution, which is shared across all partitions ^29^.

The third and final data set consists of 300 coat protein gene sequences of rice yellow mottle virus (RYMV), sampled from East to West Africa over a 46-year period from 1966 to 2012 ^31^. We set a skygrid cutoff value of 200 years, and we estimate the log population size for 100 time intervals. We assume an HKY nucleotide substitution model ^27^ for the first and second codon positions combined, and another for the third codon position, and we assume among-site rate variation ^28^ on each of these two partitions. Finally, we again assume an uncorrelated relaxed molecular clock with an underlying lognormal distribution ^29^.

### Analysis

All analyses were performed using BEAST 1.10.5 ^13^, along with the high-performance BEAGLE 3.2.0 library ^15^ for computational efficiency. Our central processing unit (CPU) analyses were performed on a single 18core Intel Xeon 6140 Skylake processor running at a clock speed of 2.3 GHz. The Ebola data set, however, necessitates the use of a powerful graphical processing unit (GPU), and these analyses were hence performed on an NVIDIA Tesla P100 SMX2 graphics card designed for scientific computing. The rabies and RYMV data sets were run for 50 million iterations, logging every 2,000 iterations, whereas the Ebola data set was run for 100 million iterations, also logging every 2,000 iterations. The posterior samples were used to construct a maximum clade credibility (MCC) tree for each data set using TreeAnnotator, discarding 10% of the samples except for the RYMV data set for which we discarded 20% of the samples.

To directly compare the performance of the different transition kernels on estimating the parameters of the Bayesian skygrid model, we performed an initial analysis that focused solely on estimating the log population size and precision parameters. To this end, we fixed the phylogeny to the MCC tree and the non-skygrid parameters to their mean posterior estimates. All data sets were analysed in BEAST for 20,000 iterations, logging every two iterations.

We evaluated the performance of the different transition kernels by computing the effective sample size (ESS) for each parameter of interest using the coda package ^32^ in CRAN R ^33^. The ESS estimates the number of in-dependent draws from the posterior distribution that an MCMC sample is equivalent to by accounting for the autocorrelation in the sample ^34^. Thus the ESS per unit time provides a measure of how efficiently a given transition kernel is sampling from the posterior distribution. We report the difference in ESS per unit time of the skygrid’s precision parameter, as well as the minimum and median ESS values of the log population size parameters.

## Results

### Inference on a fixed phylogeny

**Figure 1.**
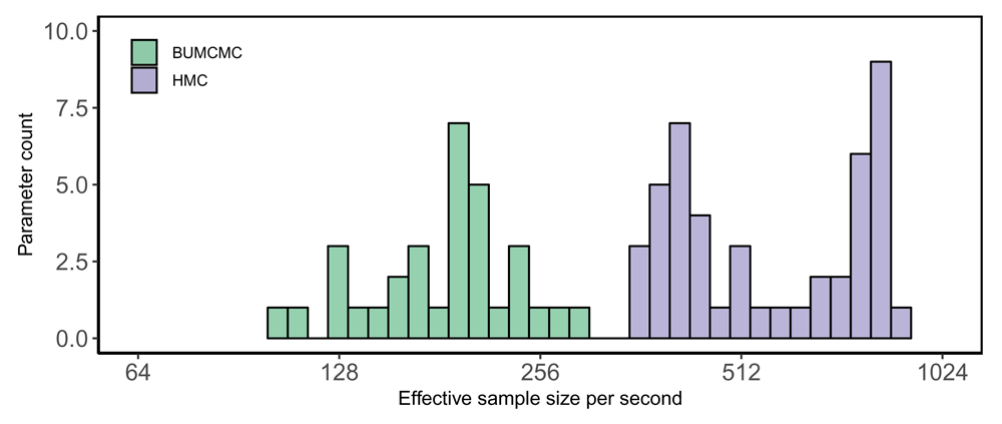
Rabies data set - fixed tree analysis. Bars correspond to the estimated effective sample size (ESS) per second averaged across five independent replicates for all log population size parameters, using the block-updating Markov chain Monte Carlo (BUMCMC) and Hamiltonian Monte Carlo (HMC) transition kernels. The height of each bar indicates the number of parameters that achieve the given ESS per second value. The HMC transition kernel improves upon the performance of the BUMCMC transition kernel by factors of 5.35 and 3.91 for the minimum and median ESS per second across all log population sizes.

We first compare the performance of the different transition kernels when solely estimating the skygrid parameters, fixing the phylogeny to the MCC tree and all other parameters to their posterior mean estimates. For the rabies data set, Figure 1 shows a pronounced improvement in performance of HMC over BUMCMC. HMC generates a 3.91-fold and 5.35-fold improvement in median and minimum ESS per second over BUMCMC for the log population sizes, and a 1.90-fold improvement in ESS per second for the precision.

**Figure 2.**
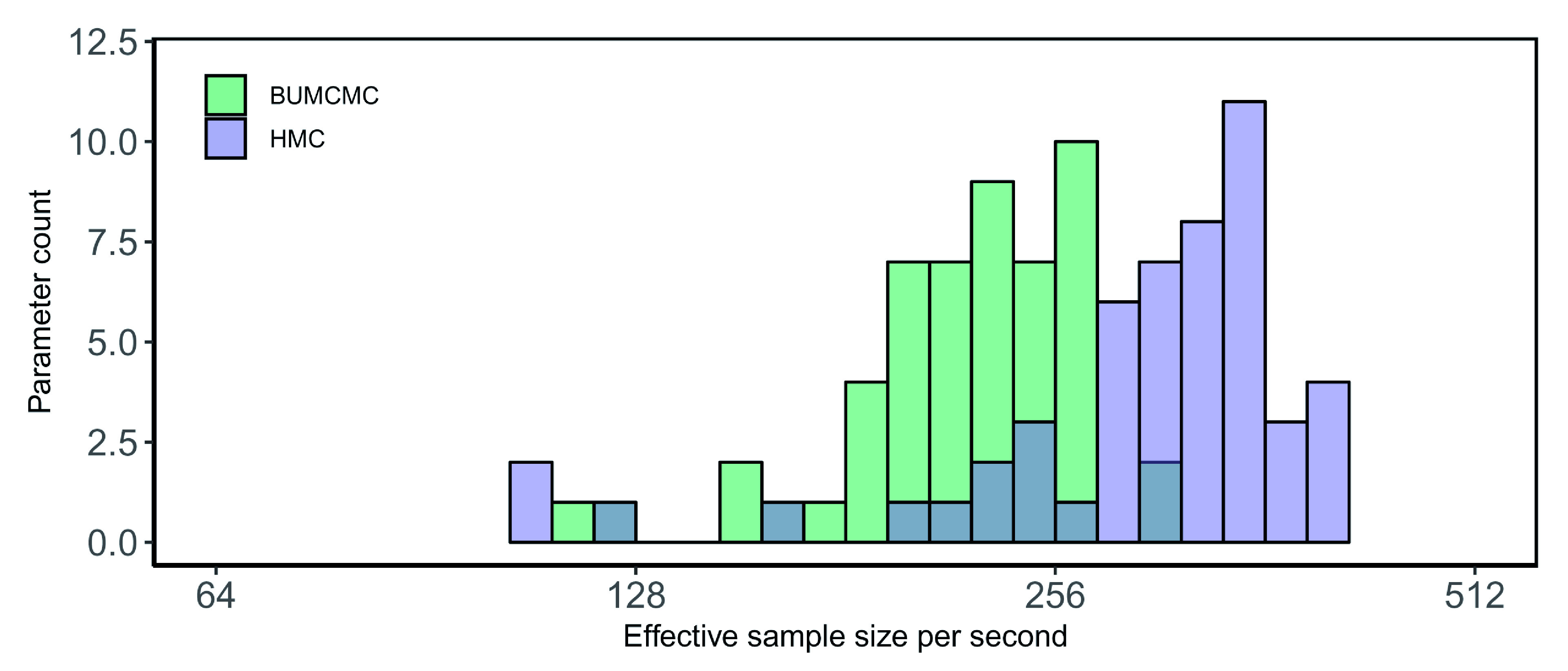
Ebola virus data set - fixed tree analysis. Bars correspond to the estimated effective sample size (ESS) per second averaged across five independent replicates for all log population size parameters, using the block-updating Markov chain Monte Carlo (BUMCMC) and Hamiltonian Monte Carlo (HMC) transition kernels. The height of each bar indicates the number of parameters that achieve the given ESS per second value. The HMC transition kernel improves upon the performance of the BUMCMC transition kernel by a factor of 1.41 for the median ESS per second but BUMCMC yields a 1.08-fold improvement over HMC for the minimum ESS per second.

**Figure 3.**
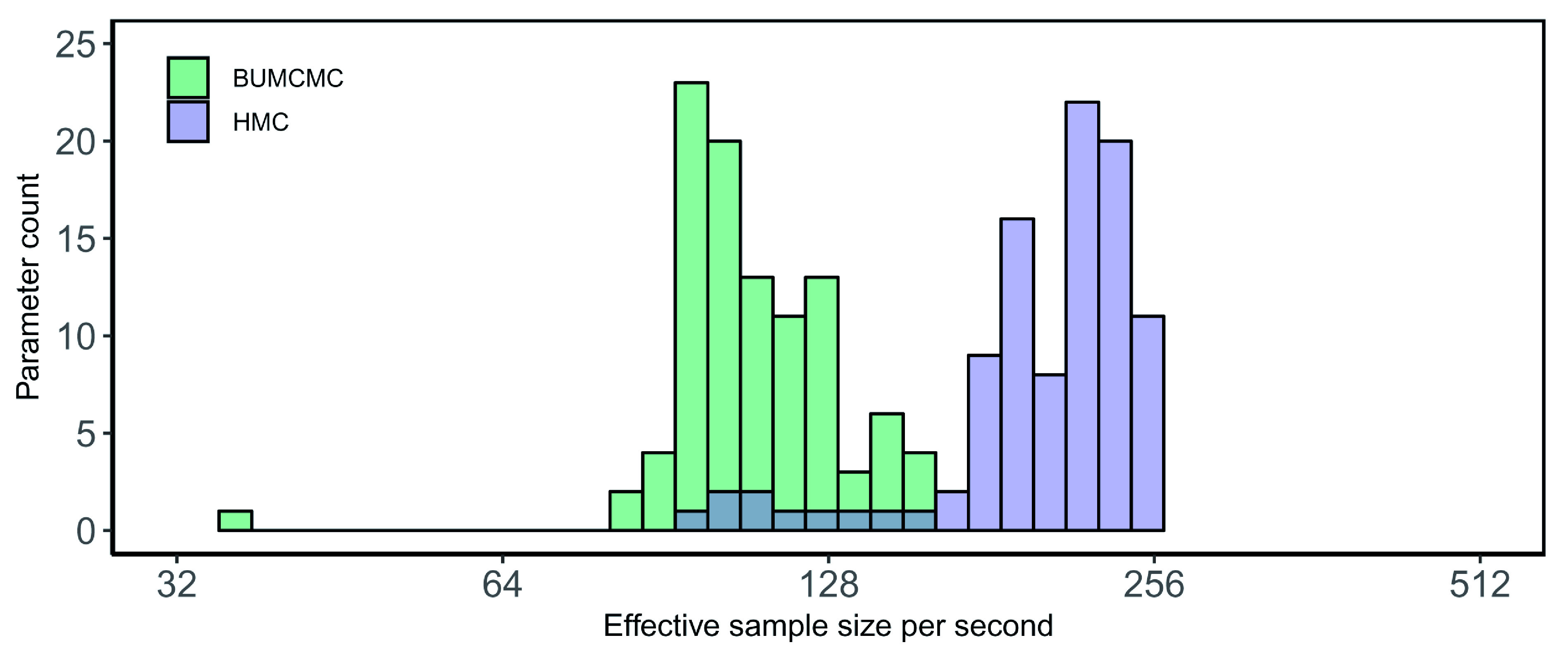
Rice yellow mottle virus data set - fixed tree analysis. Bars correspond to the estimated effective sample size (ESS) per second averaged across five independent replicates for all log population size parameters, using the block-updating Markov chain Monte Carlo (BUMCMC) and Hamiltonian Monte Carlo (HMC) transition kernels. The height of each bar indicates the number of log population size parameters that achieve the given ESS per second value. The HMC transition kernel improves upon the performance of the BUMCMC transition kernel by factors of 2.05 and 2.77 for the median and minimum ESS per second, respectively.

Figure 2 and Figure 3 show the performance improvements brought about by HMC over BUMCMC for the EBOV and RYMV data sets, respectively. For the Ebola data set, HMC yields a 1.41-fold performance increase in median ESS per second for the log population sizes and a 5.47-fold increase for the precision over BUCMC, but the latter offers a 1.08-fold improvement in minimum ESS per second over the former. Finally, for the RYMV data set, compared to BUMCMC the HMC transition kernel yields a 2.05-fold and 2.77-fold relative speedup in ESS per second in terms of the median and minimum ESS per second of the log population sizes respectively, while generating a 3.67-fold relative speedup in ESS per second for the precision. In conclusion, when focusing solely on estimation of the skygrid’s parameters on a fixed phylogeny, HMC consistently reports substantial performance increases in estimating these parameters, with the magnitude of these improvements being dependent on the specific data set and the balance between the number of population sizes and the number of taxa available in the data set.

### Joint inference

**Figure 4.**
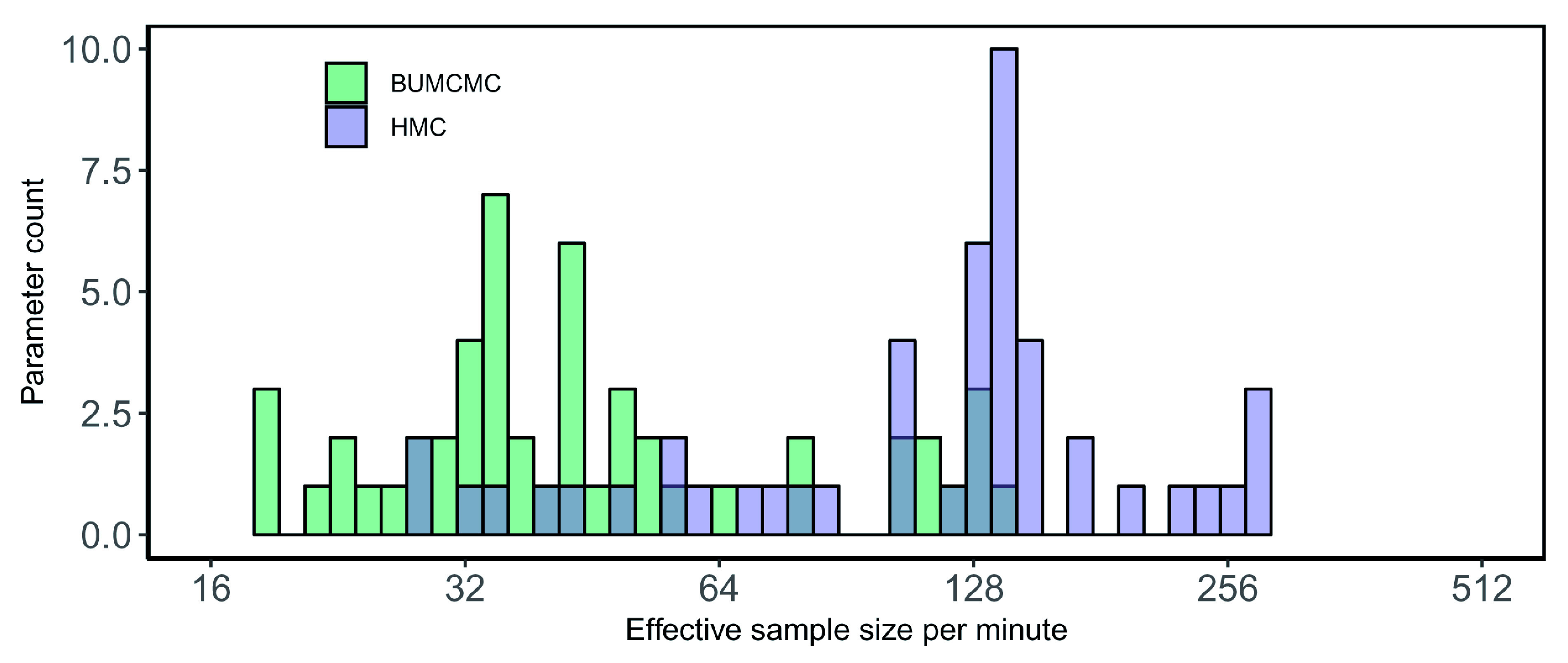
Rabies data set analysis using the skygrid coalescent model on a central processing unit (CPU). Bars correspond to the estimated effective sample size (ESS) per second averaged across five independent replicates for all log population size parameters and the precision parameter, using the block-updating Markov chain Monte Carlo (BUMCMC) and Hamiltonian Monte Carlo (HMC) transition kernels. The height of each bar indicates the number of skygrid parameters that achieve the given ESS per minute value. The HMC transition kernel improves upon the performance of the BUMCMC transition kernel by factors of 3.38 and 1.51 for the median and minimum ESS per minute, respectively, while a 3.99-fold improvement for the precision was generated.

The improvements under HMC that we observe in analyses that solely infer skygrid parameters are mirrored in more comprehensive analyses that jointly infer the phylogeny and all other model the most common use case for this model. Figure 4 shows a substantial performance increase in ESS per minute of HMC over BUMCMC for the rabies data set. Employing HMC results in a 3.38-fold and 1.51-fold relative speedup in the median and minimum ESS per minute, respectively – over BUCMC for the log population sizes and a 3.99-fold speedup for the precision.

**Figure 5.**
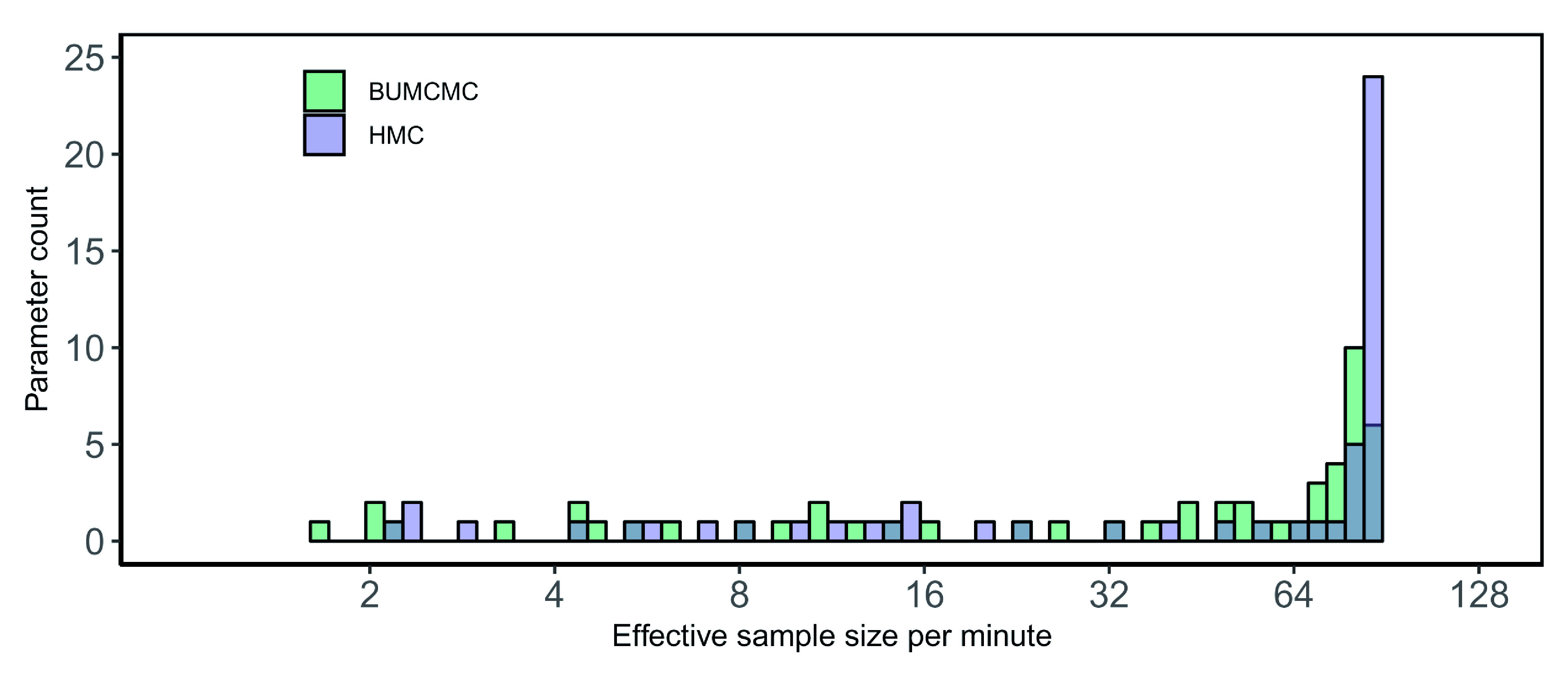
Ebola virus data set analysis using the skygrid coalescent model on a graphical processing unit (GPU). Bars correspond to the estimated effective sample size (ESS) per second averaged across five independent replicates for all log population size parameters and the precision parameter, using the block-updating Markov chain Monte Carlo (BUMCMC) and Hamiltonian Monte Carlo (HMC) transition kernels. The height of each bar indicates the number of skygrid parameters that achieve the given ESS per minute value. The HMC transition kernel improves upon the performance of the BUMCMC transition kernel by factors of 1.48 and 1.35 for the median and minimum ESS per minute, and a factor of 1.56 for the precision.

**Figure 6.**
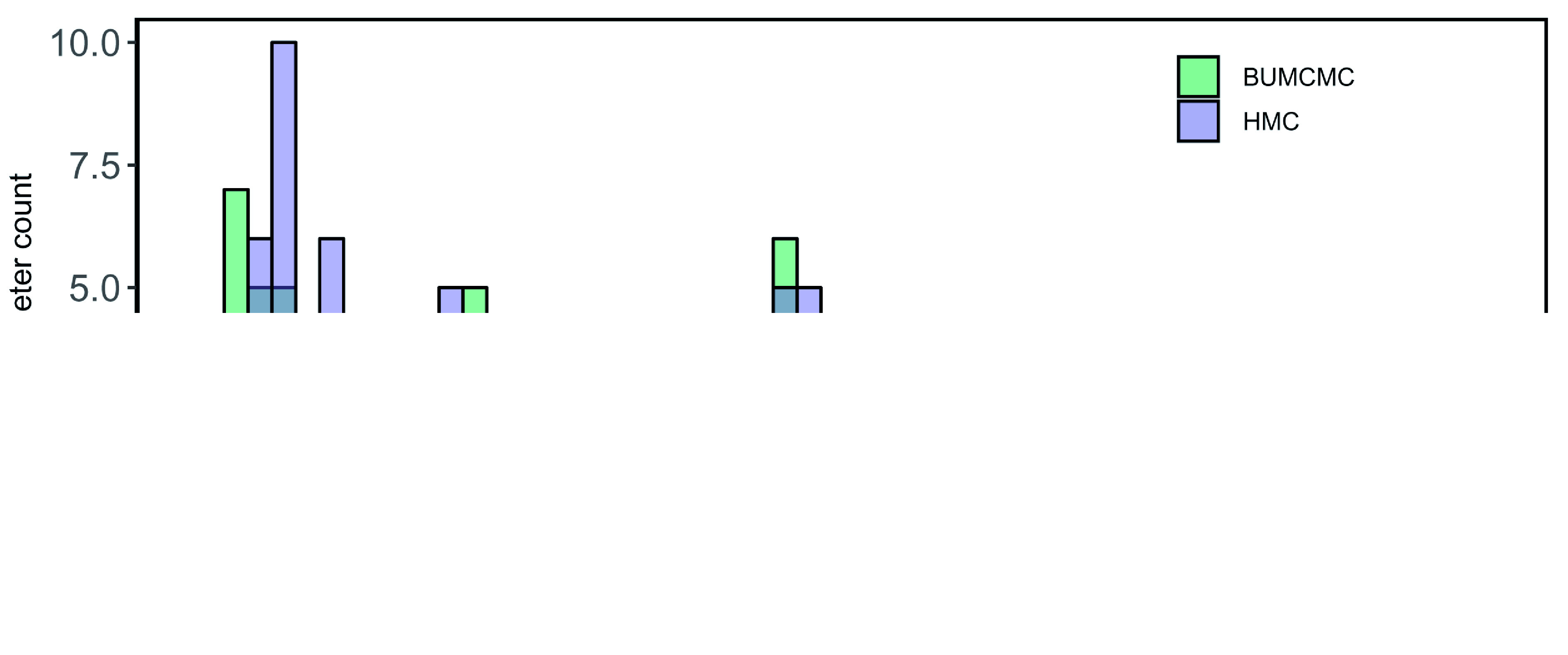
Rice yellow mottle virus data set analysis using the skygrid coalescent model on a central processing unit (CPU). Bars correspond to the estimated effective sample size (ESS) per hour averaged across five independent replicates for all log population size parameters and the precision parameter, using the block-updating Markov chain Monte Carlo (BUMCMC) and Hamiltonian Monte Carlo (HMC) transition kernels. The height of each bar indicates the number of skygrid parameters that achieve the given ESS per hour value. The HMC transition kernel improves upon the performance of the BUMCMC transition kernel by factors of 1.07 and 1.45 for the minimum ESS per hour of the log population sizes and the precision, respectively. In turn, BUMCMC yield a 1.08-fold improvement over HMC for the median ESS per hour of the log population sizes.

For the EBOV data set, Figure 5 also shows a clear performance benefit of the HMC transition kernel over BUCMC, reporting a 1.48-fold and 1.35-fold speedup in median and minimum ESS per minute for the log population sizes and a 1.56-fold speedup for the precision. For the RYMV data set, the performance improvements of HMC over BUMCMC are more modest, with Figure 6 showing nearidentical performance between HMC and BUMCMC. HMC yields a 1.07-fold speedup in terms of minimum ESS per hour over BUMCMC for the log population sizes, whereas BUMCMC in turn yields a 1.08-fold improvement in median ESS per hour over HMC. Estimation efficiency of the skygrid’s precision is however 1.45-fold faster using HMC compared to BUMCMC.

## Discussion

Coalescent-based models that relate population dynamics to genealogical shapes are central to phylogenetic and phylodynamic inference. The increasing availability of large molecular sequence data sets is testing the limits of current Bayesian phylogenetic inference software, and estimation procedures that can scale efficiently are critically important. In statistics, HMC has emerged as one of the most powerful approaches in MCMC sampling, opening the door to more efficient exploration of high-dimensional distributions through accounting for the distribution’s geometric structure. Here, we have evaluated the utility of HMC for posterior inference for the skygrid coalescent model.

In analyses of rabies, Ebola virus, and RYMV data sets, we observe that HMC consistently outperforms the standard skygrid BUMCMC transition kernel in terms of more efficiently generating effectively independent samples of skygrid model parameters. For some data sets and model parameters, HMC is over five times as efficient as BUMCMC. Such gains in efficiency are especially valuable considering the increasingly important role that phylodynamic inference methods have assumed in real-time analysis of outbreak dynamics ^35^. Advances in portable genomic sequencing capabilities have enabled sequencing during outbreaks in close to real-time ^36^, and phylodynamic inferences can potentially inform outbreak response efforts by public health apparatuses, provided that such inferences are made available in a timely manner.

Further performance improvements for the proposed HMC transition kernel may be achieved by replacing the standard choice of **I** with a more informative matrix **M**, which is equivalent to preconditioning the posterior distribution by transforming the parameters ****Φ****. Girolami and Calderhead ^37^ suggest the negative Hessian as an alternative that better accounts for the target distribution’s underlying geometry. However, such an approach is computationally prohibitive, necessitating numerical integrators that require several iterations of calculating and inverting the mass matrix at each step. Recently, however, Ji *et al.* ^24^ put forth a method to adaptively tune the mass matrix to estimate the expected Hessian averaged over the posterior distribution and avoid excessive computational burden. Extending the HMC approach for the skygrid model by tuning the mass matrix is the subject of future work.

The performance improvements that we see under HMC are also very encouraging in the context of further development and use of HMC methods in Bayesian phylogenetics and phylodynamics. It is particularly notable that a standard HMC implementation outperforms the BUMCMC transition kernel that was specifically designed for GMRF models and relied on and exploited many aspects of the skygrid model structure. In that regard, the performance improvements reported here are not directly comparable to those in the work of Ji *et al.* ^24^, who reported massive performance gains when comparing HMC transition kernels to simple univariate transition kernels. This illustrates the power of HMC and its potential for allowing statisticians to avoid developing estimation procedures that, while efficient, may only apply to a narrow range of models. Extensions to standard HMC that seek to improve sampling efficiency by better accounting for the posterior distribution’s geometric structure ^21, 37, 38^ and optimizing path lengths for numerical solutions of Hamiltonian dynamics ^39, 40^ offer further improvements and illustrate the need for continued development.

## Data availability

### Underlying data

Zenodo: GuyBaele/skygrid_hmc_data: First release of BEAST XML files for skygrid+HMC. https://doi.org/10.5281/zenodo.3715408 ^41^

This project contains the following underlying data:

- Rabies dataset BEAST 1.10.5 XML files using both BUMCMC and HMC transition kernels
- Ebola dataset BEAST 1.10.5 XML files using both BUMCMC and HMC transition kernels
- RYMV dataset BEAST 1.10.5 XML files using both BUMCMC and HMC transition kernels

Data are available under the terms of the Creative Commons Zero "No rights reserved" data waiver (CC0 1.0 Public domain dedication).

The rabies virus sequences were originally published by Biek *et al.* ^26^, DOI: https://doi.org/10.1073/pnas.0700741104. The Ebola virus sequences were originally published by Dudas *et al.* ^30^, DOI: https://doi.org/10.1038/nature22040, and the subset analysed here was created in Hill and Baele ^14^, DOI: https://doi.org/10.1093/molbev/msz172. The rice yellow mottle virus sequences were originally published by Trovão *et al.* ^31^, DOI: https://doi.org/10.1093/ve/vev016.
