# Supplementary material for: Hamiltonian Monte Carlo sampling to estimate past population dynamics using the skygrid coalescent model in a Bayesian phylogenetics framework
Source: Wellcome Open Res. 2020 Mar 30;5:53. [Version 1] doi: 10.12688/wellcomeopenres.15770.1 (PMC7463299; doi:10.12688/wellcomeopenres.15770.1)
Supplement: Peer review history

# Peer review: Reviewer response for version 1

**DOI:** 10.21956/wellcomeopenres.17296.r39859

**Published:** 2020-09-01

**Authors:** Nicola Müller

**Affiliations:** 1 Fred Hutchinson Cancer Research Center, Seattle, WA, USA

Baele *et al*. introduce a Hamiltonian Markov Chain Operator to more efficiently explore non-parametric effective population size dynamics in BEAST. While not completely novel, this presents a useful addition to users of BEAST. The paper is overall well written and particularly the comparison of ESS per second on fixed trees nicely shows the advantages of using an HMC operator. The comparison when jointly inferring the trees alongside the Ne dynamics are a bit less convincing. I would suggest to add some test about what is limiting convergence in this scenario and to compare the performance of the HMC operator to the adaptive multivariate gaussian operator implemented by some of the same authors. Also, the convergence criteria should probably be changed (at least for the joint inferences).

 Major comments:

- 5 runs are too few to compare ESS values across runs. Especially, to put an actual number on the fold increase of ESS values between the different operators. I would also argue that the ESS for a log Ne is not the most important ESS and would instead use the ESS of the posterior probabilities, in particular for the joint inferences. I would assume that the last few time intervals of the skygrid are often essentially sampling from the prior. So, by comparing ESS values of individual log Ne’s, at least partly, the comparison is in how good the two operators are in sampling from the prior. There should also be some comparison of variance of ESS between runs to compare how reliable the fold increases are.
- The major limitation of joint analyses with the phylogenetic trees are typically the tree inference themselves. Operations on the trees are often also substantially more computationally expensive, while operations on the Ne’s are fairly cheap (recomputing tree likelihoods is more expensive than recomputing the coalescent probability). This, in turn, would suggest that the weights of inefficient operators on the Ne trajectories could simply be increased without leading to a lot of extra computation time. I would suggest adding an experiment where the weights of the Ne operators are increased and ESS per unit of time are compared again.
- I’ve used the adaptive multivariate gaussian operator by Beale *et al.* for skyline type analyses. This one additionally provides the advantage of “learning” the correlation structure with other parameters (e.g. the clock rate). Is there any advantage of using the HMC approach instead of using the adaptive multivariate gaussian operator?

Minor comments:

- “In a Bayesian framework, coalescent models function ….” (Long and heavy sentence).
- “the skygrid can incorporate data from multiple unlinked genetic loci” same is true for EBSP.
- “For the rabies data set, Figure 1 shows a pronounced improvement” would replace “pronounced”.

Is the rationale for developing the new method (or application) clearly explained?

Yes

Is the description of the method technically sound?

Yes

Are the conclusions about the method and its performance adequately supported by the findings presented in the article?

Partly

If any results are presented, are all the source data underlying the results available to ensure full reproducibility?

Yes

Are sufficient details provided to allow replication of the method development and its use by others?

Yes

Reviewer Expertise:

Bayesian Phylogenetics and Phylodynamics.

I confirm that I have read this submission and believe that I have an appropriate level of expertise to confirm that it is of an acceptable scientific standard, however I have significant reservations, as outlined above.

# Peer review: Reviewer response for version 1

**DOI:** 10.21956/wellcomeopenres.17296.r39664

**Published:** 2020-08-13

**Authors:** Shiwei Lan

**Affiliations:** 1 Arizona State University, Tempe, AZ, USA

The submitted manuscript, *Hamiltonian Monte Carlo sampling to estimate past population dynamics using the skygrid coalescent model in a Bayesian phylogenetics framework*, applies Hamiltonian Monte Carlo (HMC) to estimate population dynamics for coalescent model in Bayesian phylogenetics.

 While it is an important research question in the field and the paper provides interesting applications, it overlaps existing work thus affects its originality.

 Gaussian approximation to HMC is not something new and has been extensively explored by, e.g. the following papers:

- Split Hamiltonian Monte Carlo (2014) ^1^
- Semi-Separable Hamiltonian Monte Carlo for Inference in Bayesian Hierarchical Models (2014) ^2^
- An efficient Bayesian inference framework for coalescent-based nonparametric phylodynamics (2015) ^3^

Though not mentioned by the submitted paper, it shares substantial similarity with ^3^, which details and optimizes the splitting strategy to the almost same model. The only thing different is that the submitted work includes genealogies in the inference which is not present in ^3^. 

^3^ does a thorough study in this topic by comparing split HMC with many popular MCMC algorithms including MALA, adaptive MALA, elliptic slice sampler, and Bayesian Skyride as well.

 The paper has the following issues.

  ('L' stands for line, and 'P' stands for page, negative number -XX means 'the last but XX'.)

**Major issues:**

- For all numeric experiments, how do their posterior estimates compare? It is not all about ESS per unit time. If the computationally light-weighted algorithm introduces more bias, or converges slowly, then we need to think of the trade off between efficiency and accuracy.
- Can you compare your method to other algorithms than BUMCMC?
- What does the blue-grey color stand for in the figures? It is not in the legend or explained.
- Did you repeat your experiment to reduce the sampling error?
- I am curious why collecting millions of samples? I understand that the estimate needs sufficient samples to reduce variance, but what is the computational cost?

**Minor issues:**

- P14L22 (below equation (1)): What is MRCA? Similarly HKY? Please consider giving the full name when you mention them for the first time.
- P5L-12 (below equation (17)): Could you please change the symbol $f$ in $\tau^*=\tau^{(n)} f$ -- it is very easy to confuse with the function $f$ as in equation (16) right above.
- P5L7: What is $\beta$ in $\phi=(\gamma,\tau,\beta)$? Do you mean $g$?

Is the rationale for developing the new method (or application) clearly explained?

Yes

Is the description of the method technically sound?

Partly

Are the conclusions about the method and its performance adequately supported by the findings presented in the article?

No

If any results are presented, are all the source data underlying the results available to ensure full reproducibility?

No

Are sufficient details provided to allow replication of the method development and its use by others?

Partly

Reviewer Expertise:

Statistical computing; Bayesian modeling; Uncertainty quantification

I confirm that I have read this submission and believe that I have an appropriate level of expertise to confirm that it is of an acceptable scientific standard, however I have significant reservations, as outlined above.

## References

[1] : Split Hamiltonian Monte Carlo. *Statistics and Computing*.2014;24(3) : 10.1007/s11222-012-9373-1 339-349 10.1007/s11222-012-9373-1

[2] : Semi-Separable Hamiltonian Monte Carlo for Inference in Bayesian Hierarchical Models. *Advances in Neural Information Processing Systems*.2014; Reference source

[3] : An efficient Bayesian inference framework for coalescent-based nonparametric phylodynamics. *Bioinformatics*.2015;31(20) : 10.1093/bioinformatics/btv378 3282-9 10.1093/bioinformatics/btv378 26093147PMC4795633

# Peer review: Reviewer response for version 1

**DOI:** 10.21956/wellcomeopenres.17296.r38402

**Published:** 2020-05-04

**Authors:** Mario dos Reis

**Affiliations:** 1 School of Biological and Chemical Sciences, Queen Mary University of London, London, UK

The majority of Bayesian inference problems in phylogenetics are not analytically tractable. This means we must use the MCMC stochastic algorithm to obtain samples from the posterior distribution. The most widely used flavour of MCMC in phylogenetics is the Metropolis-Hastings sampler, which shows random walk behaviour and produces MCMC chains with high autocorrelation and high rejection rates, specially in problems with large number of parameters. The Hamiltonian Monte Carlo (HMC) sampler uses physics simulations to generate efficient MCMC proposals. In the HMC method, a particle is shot across the transformed posterior surface and the laws of physics are used to calculate the trajectory of the particle. The posterior is transformed in such a way that the particle becomes trapped in regions of high probability. Furthermore, by pushing the particle far enough, we ensure the particle lands far from its starting position. Thus, the HMC sampler tends to generate MCMC chains with high acceptance rates and low autocorrelation, making the sampler very efficient.

 In phylogenetics, HMC has been used to estimate amino acid substitution matrices (Zhao *et al*. 2016) ^1^, to explore tree space (Dinh *et al*. 2017) ^2^ and to explore proposal efficiency in a two-species problem (Thawornwattana *et al*. 2018) ^3^. Recently, preprints have appeared suggesting further applications (Ji *et al*. 2019; Bastide *et al*. 2020) ^4^^,^^5^. Here, Baele *et al*. extend the application of HMC to sampling population parameters from the coalescent process in phylogenies.

 This is a well thought out, novel application of HMC to phylogenetics. Baele *et al*. show that the new HMC sampler outperforms their previous BUCMC approach by roughly 2-5x when tested on several virus datasets. That is, HMC will generate effective sample sizes (ESS) for the coalescent parameters in up to less than one-fifth of the time required by the previous sampler (depending on the dataset). These are very impressive improvements, particularly given that phylogenomic analysis on large datasets can take several days to compute. But even more importantly, it appears that further improvements to the new HMC sampler can still be achieved: The authors used the identity matrix as the mass matrix of the particle. As they acknowledge, different choices for the mass matrix should be explored. In my experience, a well fine-tuned mass matrix can provide dramatic improvements on the efficiency of HMC samplers.

Is the rationale for developing the new method (or application) clearly explained?

Yes

Is the description of the method technically sound?

Yes

Are the conclusions about the method and its performance adequately supported by the findings presented in the article?

Yes

If any results are presented, are all the source data underlying the results available to ensure full reproducibility?

Yes

Are sufficient details provided to allow replication of the method development and its use by others?

Yes

Reviewer Expertise:

Bayesian phylogenetics, molecular clock, divergence time estimation.

I confirm that I have read this submission and believe that I have an appropriate level of expertise to confirm that it is of an acceptable scientific standard.

## References

[1] : Bayesian Analysis of Continuous Time Markov Chains with Application to Phylogenetic Modelling. *Bayesian Analysis*.2016;11(4) : 10.1214/15-BA982 1203-1237 10.1214/15-BA982

[2] : Probabilistic path Hamiltonian Monte Carlo. *Proceedings of the 34th International Conference on Machine Learning*.2017;1009-1018

[3] : Designing Simple and Efficient Markov Chain Monte Carlo Proposal Kernels. *Bayesian Analysis*.2018;13(4) : 10.1214/17-BA1084 1037-1063 10.1214/17-BA1084

[4] : Gradients do grow on trees: a linear-time O(N)dimensional gradient for statistical phylogenetics. *arXiv:1905.12146*.2019;10.1093/molbev/msaa130PMC753061132458974

[5] : Efficient Bayesian Inference of General Gaussian Models on Large Phylogenetic Trees. *arXiv:2003.10336*.2020;
